# Farewell Dinner to Dr. Conolly on His Leaving Warwickshire

**Published:** 1839-07

**Authors:** 


### FAREWELL DINNER TO DR. CONOLLY,

### ON HIS LEAVING WARWICKSHIRE.

IT is always an agreeable duty with us to record proceedings of a public character which do honour to our profession or to its members; this duty is doubly pleasant when, as on the present occasion, such proceedings have reference to a most dear and much valued friend. The official relation in which we have so long stood to Dr. Conolly being broken by his appointment to HANWELL, we might be supposed at liberty to indulge our feelings by stating how much we have profited by his kind assistance during our long and happy association, and how deeply we prize the friendship which we trust no change of place or of fortune will ever interrupt; but we are restrained by an apprehension of giving pain to that modesty which forms as marked a feature in the character of our friend as the great talents and virtues by which it is accompanied. Our regret at losing the cooperation of Dr. Conolly in the editorial department of this Journal—and we are happy in believing, in the *editorial* department only—is counterbalanced by the conviction that he is at length placed in a situation peculiarly suited to his tastes and talents, and by the confident belief that that most important department of medicine to which his attention will be now almost exclusively devoted, will derive very great advantages from his future labours.

We extract the following account literally from a Warwickshire paper, and regret that our limits permit us to insert only a small part of the original Report:

We have very great pleasure in publishing a condensed report of the proceedings of this interesting meeting, which took place at the Town Hall, Stratford-on-Avon, on Monday last. The entertainment originated with the members of the Shakspearean Club, who were anxious, without any formal or public notification, to testify their deep sense of Dr. Conolly's zealous attention to the interests of that society. We were pleased to observe but one feeling pervading the assembly, viz., that of satisfaction that so honorable an appointment as that of Resident Physician of the Middlesex Lunatic Asylum, should have devolved upon one, whose accomplishments as a scholar and whose virtues as a gentleman had long since endeared him to a numerous circle of friends and admirers. It will afford some satisfaction to every true Shakspearean resident in this neighbourhood to know that, although removed from the sphere of his early and active exertions in its behalf, the society may encourage a hope of occasionally finding Dr. Conolly a participator in their annual tributary honours to the memory of Shakspeare."

After the usual toasts the Chairman, W. J. Harding, Esq., of Baraset House, observed, "that the next toast was intimately connected with the object for which they were assembled. Having been requested by the members of the Shakspearean Club to preside on that occasion, he must entreat their indulgence for any inadequacy which might appear in the performance of the duty he had undertaken. There were many members present who would address the meeting, and do that justice to the subject of the toast which he candidly acknowledged he felt himself incompetent to perform. He would invite them to drink the health of their worthy and talented guest, Dr. Conolly. Filling the situation which he then occupied, he felt himself called upon to express, however inadequately, the sentiments of the Shakspearean Club towards that most excellent and respected man; and, although he might fail in expressing them in full force, it was not difficult to know what they were; they were to be found in one common feeling of admiration, gratitude, and respect. (Great applause.) Had that meeting been considered a public one, neither that room nor any other in the neighbourhood would have been sufficiently extensive to admit the numerous assemblage of friends and admirers of their respected guest, who would have dined with the society on such an occasion; but the meeting was confined to the members of the Shakspearean Club, and a few of their friends, resident in Stratford or its immediate vicinity, who were anxious to do honour to the public and private virtues of Dr. Conolly: and was to be regarded rather as the expression of the social and kind feelings of the club, than any public demonstration of that respect which would have awaited Dr. Conolly, had the assembly assumed a more extended character. There was no gentleman merited better the honour then conferred upon him; which, from the modesty which had ever been his greatest characteristic, would be more agreeable to him than any more ostentatious mode of expressing their feelings towards him. Notwithstanding they all regretted that the present occasion was to be regarded as a leave-taking, they had the satisfaction of knowing that Dr. Conolly had recently received an appointment in every way suitable to his wishes, and which might, indeed, be looked upon as a fitting reward for his talents, and a public recognition of those merits which had so long and so highly been estimated by his friends. The gentlemen with whom the appointment rested, viz., the Magistracy of Middlesex, had, in his (the Chairman's) opinion, conferred great honour upon themselves in selecting a gentleman who had devoted, during a long series of years, so much attention to that particular branch of medical science, and was, in every way, eminently qualified to protect and relieve the unfortunate individuals whose distressing maladies might place them under his care. In reference to the Shakspearean Club, they could not forget how much they were indebted to the residence of Dr. Conolly amongst them for a promotion of that and other institutions upon which they now set a high value. During his residence in the town of Stratford, he became universally beloved by all classes of society and the inhabitants, of which there could be no more gratifying proof than the presentation of a piece of plate by Sir Gray Skipwith, in the name of his townsmen and friends. He (the Chairman) was not in the kingdom at that time, but he recollected that, on his return, he universally heard Dr. Conolly spoken of as a skilful and benevolent physician, and an indefatigable friend to the poor and afflicted. He afterwards witnessed the Dr.'s great and assiduous attention in promoting the adornment of the Chancel, and the preservation of the remains of Shakspeare and his family. Nothing could exceed his constant attention at the meetings of the Committee, where his services were of the most invaluable character. Dr. Conolly had left Stratford with the regret of all classes, and at Warwick and Birmingham, where he had since resided, he was distinguished for his ardent promotion of similar objects, and was equally and generally beloved. Indeed, he was a man of such commanding talent and philosophic character, of so much modesty of demeanour and amiability of disposition, that it was impossible that he could be otherwise than admired, respected, and beloved. In fact, whether as a public man or as a private individual, few could approach him. (Great cheering.) In these times of improvement, when time and space were so much abridged, he could not but cherish the hope that they should often see Dr. Conolly at their annual celebration of the Poet's birthday. How often had they been rendered happy and delighted, when the Doctor had, from that chair, expatiated on the character and writings of the Immortal Bard, with all his admirable taste and eloquence of language; and he could not think of those occasions without still wishing that they might often have the opportunity of seeing him amongst them on the 23d of April. (Cheers.) He proposed the health of Dr. Conolly, with three times three: might he live long to enjoy an appointment for the duties of which he was so admirably qualified."—The toast was drank amidst the most deafening applause.

"Dr. Conolly rose to return thanks for the honour done to him. When he received the kind invitation of the club, his reply had been that he had known the members too long, and had experienced their kindness too often, to require any formal manifestation of it. But he must confess that the manner in which they met him that day was deeply gratifying to him. They had known him long and intimately; through good and evil report they had been his steadfast friends; he had come among them, many years ago, a stranger to them all; the best, the happiest, the most energetic of his years had been passed among or near them; his hair (he might literally say) had grown gray among them; his children had grown up among them; and well might he prize the cordial and honest testimony of those to whom so much of his life and conduct was necessarily known. And, he must add, no man could practise the profession of medicine in any community for many years, without witnessing such changes and chances in the families of his friends and neighbours, and so many scenes calculated to call forth the deepest and strongest affections, as to awaken and daily strengthen in his mind those bonds of peace and charity which were the most suitable to those engaged in the same pilgrimage. Therefore it became doubly grateful to him to hear the kind accents in which they were pleased to mention his humble name and services, particularly as expressed so cordially, so courteously, but he feared, as regarded the object of them, so undeservedly, by their esteemed chairman. In that very room, sixteen years ago, he had met several of the inhabitants of the town and neighbourhood, for the purpose of instituting the dispensary. Many of its original supporters now sleep in the grave; but it was cheering to see that, although the individual actors in charitable, as in other works, must fade and decay, the work of charity itself went on and prospered. The dispensary had grown into an infirmary, which would be the means of affording important aid to the sick poor of the town and neighbourhood for ages to come. On another occasion, eleven years since, he had with much satisfaction assisted in that room in forming a Society for Reading and Lectures, which he knew had already been useful, and which he hoped would hereafter become much more so. In a neighbouring town he had had the gratification of being one of those who assisted to form the Warwickshire Natural History Society, the great success of which was to be ascribed to the extraordinary exertions of a few scientific individuals, to whom he should always feel very grateful, aided by the general interest and patronage of the inhabitants of the county. The pleasure he derived from contemplating these and other provincial institutions with which he had been accidentally connected arose not so much from their actual condition as from a belief that they were only rudimental fragments of future provincial academies or colleges, the advantages of which, unknown in the provinces of England, had long been enjoyed in other countries, by no means rivalling this country in other particulars. The first humble labourers in that rich field might then be forgotten, but the seed they planted would grow and flourish, and diffuse precious fruit throughout the land. (Cheering.) Again, looking back on Stratford recollections, he was reminded how many times he had in that room met many of them, deeply interested like himself, in devising the means of preserving the venerable fabric wherein reposed the sacred ashes of Shakspeare; to possess which was the boast of Stratford, and might well be the envy of the world. (Renewed cheering.) It delighted him to reflect that out of those efforts sprung the great effort now making by the inhabitants to preserve the whole of their beautiful parish church from decay; and within its walls, for countless centuries, he trusted their descendants would assemble to offer a pure worship, passing by, not ungratefully, the tombs of those who prevented the whole building falling into ruins. He never forgot that these designs began and were fostered in the club; and it was because the club had ever entertained such useful and liberal designs, and had ever promoted friendly feelings among the townspeople, and encouraged a temperate and honest conviviality, that he had at all times felt pleasure in meeting the members and contributing his best assistance to them. When now reviewing, as all must occasionally do, a distinct portion of his life, and asking, as all must occasionally ask, the meaning of this swiftly-passing dream, feeling that one passage of it was closing with this evening, and wishing to close it candidly and fairly with them, and peaceably with his own conscience, he felt that to one charge they might possibly think his conduct open; for if he had been so kindly treated in Warwickshire, it might be thought he ought to have made a fortune, and remained among them. His answer to this was, that to make a fortune, a physician required not only a larger population around him, but a habit of pursuing his profession a little more eagerly than he had perhaps been disposed to do; for he very early found out that much consideration was due from a physician not only to the squalid poor, but to many persons of decent and respectable station, to whom sickness brought many sacrifices, seldom avowed, but too clearly seen by a humane observer. He must add that he had a firm, although not a presumptuous trust in Providence; and if he could leave his children the legacy of an unblemished fame, he should have a better hope of their welfare and happiness than if he left them the largest fortune that grasping avarice could extort from suffering poverty. (Great applause.) And this subject reminded him that there was one topic, never touched upon before by him, because he felt that he could not heretofore touch upon it with propriety and dignity, and yet on which, now that it could no longer affect his interest, he would beg leave to say a few, a *very* few words. He knew that a belief had prevailed in the neighbourhood, how originating and how spread about he neither knew nor heeded, that on leaving Stratford he had made his friends there the subject of a gainful bargain, and on returning to Warwickshire had contravened the terms of that bargain. All the evil which this report could do him, or was intended to do him, was now done. It would no more affect him. He would only say that *there was not one word of truth in it from beginning to end.* Those around him well *knew* that it was not true; that it *could not* be true; that such conduct was abhorrent to his nature, and contradicted by his whole life. (Applause.) He wished to make no accusations; he would utter no reproaches; he fully and sincerely forgave those who had done him undeserved injury; and he never meant to allude to the subject more: but if such had been his conduct, he should have ill deserved, and could not have obtained, the favour and good opinion of his own profession, which he felt had ever attended him, and for which, strongly and widely expressed, and without his soliciation, he was solely indebted for the appointment which was the occasion of his leaving them. To possess and to deserve the esteem of the members of the medical profession, and to be an honorable member of it, had ever been, and would ever be, the first worldly object of his care, and the highest object of his pride. By the diligent exercise of the duties and virtues of that profession, as far as was in his power, he should ever seek his chief happiness, and must hope to secure honorable reputation." &c.

---

